# Maternal catalase supplementation regulates fatty acid metabolism and antioxidant ability of lactating sows and their offspring

**DOI:** 10.3389/fvets.2022.1014313

**Published:** 2022-11-23

**Authors:** Tiantian Zhou, Bei Cheng, Lumin Gao, Fengyun Ren, Guanglun Guo, Teketay Wassie, Xin Wu

**Affiliations:** ^1^CAS Key Laboratory of Agro-Ecological Processes in Subtropical Region, Institute of Subtropical Agriculture, The Chinese Academy of Sciences, Changsha, China; ^2^Hunan Co-Innovation Center of Safety Animal Production, College of Animal Science and Technology, Hunan Agricultural University, Changsha, China; ^3^Tianjin Institute of Industrial Biotechnology, Chinese Academy of Sciences, Tianjin, China

**Keywords:** catalase, antioxidant, fatty acid, lipid metabolism, suckling piglet, sows

## Abstract

**Introduction and methods:**

As a crucial antioxidant enzyme, catalase (CAT) could destroy the cellular hydrogen peroxide to mitigate oxidative stress. The current study aimed to investigate the effects of maternal CAT supplementation from late gestation to day 14 of lactation on antioxidant ability and fatty acids metabolism with regard to the sow-piglet-axis. On day 95 of gestation, forty sows were divided into control (CON) group (fed a basal diet) and CAT group (fed a basal diet supplemented with 660 mg/kg CAT), the feeding experiment ended on day 14 of lactation.

**Results:**

The lactating sows in the CAT group produced more milk, and had higher antioxidant enzymes activity including POD and GSH-Px (*P* < 0.05), lower content of serum LDL as well as plasmic C18:3n3 content (*P* < 0.05). Additionally, maternal CAT supplementation improved offspring's body weight at day 14 of nursing period and ADG (*P* < 0.05), and regulated the antioxidant ability as evidenced by decreased related enzymes activity such as T-AOC and CAT and changed genes expression level. It significantly affected lipid metabolism of suckling piglets manifested by increasing the serum ALT, CHOL, and LDL (*P* < 0.05) level and modulating plasma medium- and long-chain fatty acids (MCFAs and LCFAs), as well as regulating the genes expression involved in lipid metabolism.

**Conclusion:**

Maternal CAT supplementation could regulate the fatty acid composition and enhance the antioxidant ability of sows and offspring during the lactating period and further promote the growth of suckling piglets. These findings might provide a reference value for the utilization of CAT as supplement for mother from late pregnancy to lactation period to promote the fatty acid metabolism of offspring.

## Introduction

The impact of maternal nutrition during gestation and lactation on the health of offspring is increasingly recognized by permanently affecting growth, function, and metabolism (1, 2). Maintaining the health of sows and piglets is essential to pig production. Particularly, piglets during the nursery phase are more susceptible to face challenges because of their immature immunity system (3), undeveloped intestinal function (4), sluggish nervous system (5), etc.

The nutrition obtained by piglets with vigorous metabolic function and high nutritional demand before feeding is mainly from sows; therefore, the physiological condition of suckling piglets during the breastfeeding stage is closely related to the nutritional metabolism of sows, especially the energy metabolism (6) and protein deposition (7) of piglets.

It is well-accepted that pregnancy is often accompanied by oxidative stress, especially after 60 days of gestation, which peaks in the third trimester of pregnancy (8). Sows during pregnancy and the lactation period are acutely susceptible to oxidative stress (9) due to the developing catabolic state (10), rapid fetal growth, mammary gland development, and milk secretion. Oxidative stress could cause damage to placental structure and function and changes in milk nutrient composition, thus negatively affecting the growth and development of the offspring (11). Therefore, treating the diet of sows during late gestation and lactation seems to be a conducive way to satisfy their own nutritional requirements and improve the growth and development of their offspring. It was believed that an appropriate dosage of antioxidant nutrients for hyper-prolific sows not only enhanced the health state of sows but also improved the suckling piglets' development (12). Consequently, there is an urge to find a green and efficient feed additive to alleviate the oxidative stress of sows. Catalase (CAT) may be considered as a functional antioxidant additive in sow's feed during the late pregnancy period.

Catalase, as one of the crucial antioxidant enzymes, could destroy the cellular hydrogen peroxide to mitigate oxidative stress, which is considered to be more effective in dominating the resistance of cells to reactive oxygen species (ROS) (13). Previous studies demonstrated that exogenous CAT supplementation could improve growth performance and alleviate liver injury in weaned piglets challenged with LPS (14). In addition, it was found that CAT could affect lipid metabolism (15). However, the impacts of adding CAT to a sow's diet on the lipid metabolism and antioxidant ability of both sows and their offspring have been poorly studied. Therefore, this study aimed to investigate the effects of maternal CAT supplementation during the perinatal period on piglets' growth performance, serum parameters related to lipid metabolism, antioxidant content, and fatty acid profiles of sows and their offspring, as well as the mRNA levels related to antioxidant enzymes and fatty acid metabolism of piglets.

## Materials and methods

### Ethical statement

All animal procedures were approved by the Review Committee of the Institute of Subtropical Agriculture at the Chinese Academy of Science and Protocol Management.

### Animals and experimental design

A total of 40 pregnant sows (gestation day 95, Large White × Landrace), with a similar backfat thickness (approximately 19 mm) and mean parity of 3–5, were randomly and equally assigned into the two dietary treatment groups (20 sows per treatment). During the feeding experiment, the sows were fed a basal diet (CON group) or a basal diet supplemented with 660 mg/kg CAT (CAT group) as described in our previous study, which revealed that 660 mg/kg CAT could decrease the intrauterine growth restriction (IUGR) rate and contribute to improving the maternal and fetal antioxidant status (16). The basal diet for sows during gestation and lactation (Table 1) was formulated to conform to the nutritional requirements recommended by the NRC (2012), as stated in our previous report (16). The CAT used in this experiment was provided by the Group of Nutrient Resources and Synthetic Biology, Tianjin Institute of Industrial Biotechnology. All animals were allowed *ad libitum* access to feed and water throughout the experimental period.

**Table 1:** Composition and nutrient levels of the basal diet (air-dry basis).

**Items,%**	**Late pregnancy**	**Lactation period**
Corn	60.6	60.50
Bean pule43	11.80	15.10
Quick burst soybeans	-	10.00
Soybean hull	15.00	2.00
Rice bran	7.00	-
CaHPO4	1.60	1.60
Limestone	1.10	1.10
Biofermented feed	-	4.00
Hamlet protein 300	-	2.50
Soybean oil	0.80	0.60
Acidifier	0.50	0.50
Sod	0.25	0.40
Lysine (70%)	0.22	-
Lysine (98%)	-	0.40
Threonine	0.10	0.15
Methionine	0.05	0.05
Tryptophan (98%)	-	0.08
NaCl	0.40	0.40
Mold inhibitor	0.08	0.10
choline chloride (60%)	0.10	0.12
Mineral premix	0.20	0.20
Vitamins premix	0.20	0.20
Total	100	100
**Nutritional level,%**		
Digestible energy (Kcal/Kg)	3,050.00	3,350.00
Crude protein	12.50	17.50
Calcium	0.90	0.90
Phosphorus	0.65	0.65
Available phosphorus	0.41	0.43
Lysine	0.68	1.15
Methionine	0.26	0.32
Threonine	0.57	0.80
Crude fiber	8.00	4.00
Crude fat	3.40	5.10

The mineral premix provided the following per kilogram of diet: copper: 185 mg, iron: 3,500 mg, manganese: 1,250 mg, zinc: 1,300 mg, selenium: 3.5 mg, iodine: 190 mg; The mineral vitamins premix provided the following per kilogram of diet: vitamin A: 6,000 IU, vitamin E: 800 mg, vitamin D3: 4,000 IU, vitamin K3: 30 mg, vitamin B1: 25 mg, vitamin B2: 25 mg, vitamin B6: 80 mg, niacin: 300 mg, vitamin B12: 0.2 mg, pantothenic acid: 200 mg, folic acid: 10 mg, biotin: 4 mg, choline: 5,000 mg; The calculated value of dietary nutrients.

The whole feeding experiment started on day 95 of gestation and ended on day 14 of lactation. Sows were housed separately in gestation pens and then transferred to individual crates with a partly slatted floor and heating lamps on day 108 of gestation. Litter size was standardized by transferring piglets on the second day of farrowing between sows of the same treatment. All the routine procedures, including tail docking, ear notching, male castration, and iron injection, were performed according to the farm's requirements. Sow's milk was the unique source of food for the suckling piglet during lactation.

### Data record and sample collection

On day 95 of pregnancy and day 14 of lactation, the backfat thickness of sows was individually measured at 6 cm from the midline of the head of the last rib using an ultrasound machine (Agroscan A 16, France) as previously described (17), and then averaged. At the end of the feeding trial, all pigs fasted overnight, and then blood samples were collected from the auricular vein into 5 ml tubes from eight sows per group. Blood was centrifuged for 10 min at 3,000 × g at 4°C to obtain serum.

The body weight (BW) of piglets was measured at 7 and 14 days after farrowing. According to milk yield calculation by Close and Cole (18), a litter would require 4 kg of breast milk for 1 kg of weight gain. From each group, six 14-days-old suckling piglets with body weight close to the average litter weight per sow were selected and then were anesthetized with an iv injection of sodium pentobarbital (50 mg/kg body weight) and bled by exsanguination. Blood samples were collected from the jugular vein in 10 ml tubes. Besides, liver and intestine samples were collected for gene expression analysis.

### Serum parameters

Serum biochemical parameters, including total protein (TP), albumin (ALB), alanine aminotransferase (ALT), aspartate aminotransferase (AST), alkaline phosphatase (ALP), total triglyceride (TG), cholesterol (CHOL), low-density lipoprotein (LDL), high-density lipoprotein (HDL), and glucose (Glu), were measured using an automated biochemistry analyzer (Synchron CX Pro, Beckman Coulter, Fullerton, CA, United States) and the commercial kits from Nanjing Jiancheng Bioengineering Institute (Nanjing, China).

### Measurement of antioxidant parameters

The serum of lactating sows and suckling piglets was used to measure the glutathione (GSH), peroxidase (POD), catalase (CAT), total antioxidant capacity (T-AOC), glutathione peroxidase (GSH-Px) activity, and malondialdehyde (MDA) content using commercially available kits (Shanghai Zhuocai, China) following the manufacturer's instructions.

### Fatty acid composition

According to our previous study (17), fatty acid contents were measured and determined using gas chromatography (GC, Agilent 6,890). The results were expressed as a percentage of total fatty acids.

### RT-qPCR analysis

The liver and intestinal mucosa (jejunum and ileum) of suckling piglets were ground into powder using liquid nitrogen and then the RNA was extracted using Trizol reagent (Invitrogen, Carlsbad, CA, United States) (19). Following the integrity and concentration being checked, the RNA was reversely transcribed into cDNA with Evo M-MLV Reverse Transcription Kit II (Hunan Aikerui Biological Engineering Co., LTD, Hunan, China) according to the kit instructions. Premix Pro Taq HS qPCR Kit II (Hunan Aikerui Biological Engineering Co., LTD, Hunan, China) was used for quantitative real-time polymerase chain reaction (RT-qPCR). The RT-qPCR was performed on Roche LightCyclerfi 480II (Roche, Basel, Switzerland) with 10 μl total volume reaction, consisting of 5 μl 2X SYBR^®^ Green Pro Taq HS Premix II, 2 μl cDNA template, 0.4 μl each of forward and reverse primers, and 2.2 μl RNase free water. The primers by which real-time PCR was performed are shown in Table 2. The relative level of mRNA expression was calculated using the 2^−ΔΔCt^ method after normalization with β*-actin* as a housekeeping gene (20).

**Table 2:** Primers used for RT-qPCR.

**Target gene**	**Accession no**.	**Nucleotide sequence of primer (5^′^-3^′^)**	**Size (bp)**
ACC	XM_021066238.1	F: GCCGAAACATCTCTGGGATA	170
		R: CTCCAGGACAGCACAGATCA	
ElVOL5	XM_021098832.1	F: TACCACCATGCCACTATGCT	102
		R: GACGTGGATGAAGCTGTTGA	
FADS1	NM_001113041.1	F: GTCACTGCCTGGCTCATTCT	155
		R: AGGTGGTTCCACGTAGAGGT	
SREBP1c	XM_021066226.1	F: GACCGGCTCTCCATAGACAA	229
		R: CCTCTGTCTCTCCTGCAACC	
FABP1	AY960623.1	F: GAGTAGCCTCATTGCCACCAT	208
		R: TGCACGATTTCCGATGTCCC	
SCD	NM_213781.1	F: AAGGAGCTGGTCAGTCGTTG	243
		R: GCTTTCGAAGCTTTGTGCCA	
PPARα	NM_001044526.1	F: GGCTTACGGCAATGGCTTCA	168
		R: CGGTCTCCGCACCAAATGA	
HSL	XM_013988600.2	F: GCCTGTTTCATTGCGTTTG	198
		R: GCCGGTGACGCTGAAAGTGGTAT	
CPT-1α	NM_001129805.1	F: GCACTGGTCCTTCTGGGATA	198
		R: GCATTTGTCCCATCTTTCGT	
ATGL	NM_001098605.1	F: ATGGTGCCCTACACGCTG	111
		R: GCCTGTCTGCTCCTTTATCC	
FATP1	XM_021076151.1	F: CCCTCTGCGTCGCTTTGATG	223
		R: GCTGCGGTCCCGGAAATACA	
FABP3	NM_001099931.1	F: CCAACATGACCAAGCCTACCACA	178
		R: ACAAGTTTGCCTCCATCCAGTGT	
GPX1	NM_214201.1	F: TGGGGAGATCCTGAATTG	184
		R: GATAAACTTGGGGTCGGT	
GPX4	NM_214407.1	F: GATTCTGGCCTTCCCTTGC	173
		R: TCCCCTTGGGCTGGACTTT	
CAT	XM_021081498.1	F: CGAAGGCGAAGGTGTTTG	370
		R: AGTGTGCGATCCATATCC	
Cu/ZnSOD	NM_001190422.1	F: CCAGTGCAGGTCCTCACTTCAATC	172
		R: CGGCCAATGATGGAATGGTCTCC	
MnSOD	NM_214127.2	F: GGACAAATCTGAGCCCTAACG	159
		R: CCTTGTTGAAACCGAGCC	
β-actin	XM_003357928.4	R: GACGGCTGGATGATGTAGTTGG	132
		R: CGGCAAGACAGAAATGACAA	

### Statistical analysis

The data were subjected to independent *t*-test analysis using SPSS software, version 22.0 (SPSS Institute, Inc., Chicago, IL, United States). Data were presented as mean ± standard error of the mean (SEM) form. Differences between mean values were compared using independent samples *t*-test and considered statistically significant at *P* < 0.05, and trends were recognized when 0.05 < *P* < 0.10.

## Results

### Growth performance and milk yield

To investigate the effects of maternal CAT supplementation on lactation sows' backfat loss and suckling piglets' growth performance, sows were fed with a basal diet or CAT supplementation diet during late pregnancy and lactation (Tables 3, 4). We previously found that maternal CAT could reduce the intra-uterine growth retardation (IUGR) rate while not significantly affecting the birth body weight of piglets (16). However, in this study, maternal CAT supplementation tended to increase BW at day 14 of the nursing period (0.05 < *P* < 0.1) and significantly increased ADG (*P* < 0.05) of suckling piglets in the CAT group compared to the CON group. The BW at day 7 of the nursing period of piglets had no significant differences between the CON and CAT groups. In addition, the CAT supplementation to sows did not affect the nutritional composition of colostrum but significantly increased the milk yield during the experiment compared to the control diet (*P* < 0.05).

**Table 3:** The effects of maternal dietary CAT supplementation on growth performance of suckling piglets and milk yield of the sow.

**Items**	**Dietary treatment**		***P*** **-value**
	**CON group**	**CAT group**	
**BW of suckling piglets**			
At day 7, kg	2.31 ± 0.12	2.52 ± 0.11	0.232
At day 14, kg	3.52 ± 0.14^b^	4.03 ± 0.19^a^	0.050
ADG, g	165.96 ± 10.50^b^	200.53 ± 7.25^a^	0.017
Milk yield, kg/d	7.15 ± 0.50^b^	8.75 ± 0.38^a^	0.027

Values are presented as mean ± SEM. Statistical significances with different superscripts were set at P < 0.05. BW, body weight. ADG, average daily gain.

**Table 4:** The effects of maternal dietary CAT supplementation on colostrum nutrient composition.

**Items**	**Dietary treatment**		***p*** **-value**
	**CON group**	**CAT group**	
Somatic cells (thousand piece/ml)	255.50 ± 50.10	267.71 ± 55.89	0.79
Milk fat (%)	0.86 ± 0.10	0.96 ± 0.08	0.43
Milk protein (%)	3.56 ± 0.17	3.56 ± 0.24	0.99
Lactose (%)	0.86 ± 0.06	0.90 ± 0.03	0.52
Solid-not-fat (%)	5.18 ± 0.19	5.20 ± 0.22	0.93
Total solid (%)	6.69 ± 0.25	6.83 ± 0.22	0.68
Urea nitrogen (mg/deciliter)	15.23 ± 0.99	16.61 ± 0.92	0.32

Values are presented as mean ± SEM.

### Serum biochemical parameters

**Figure 1:**
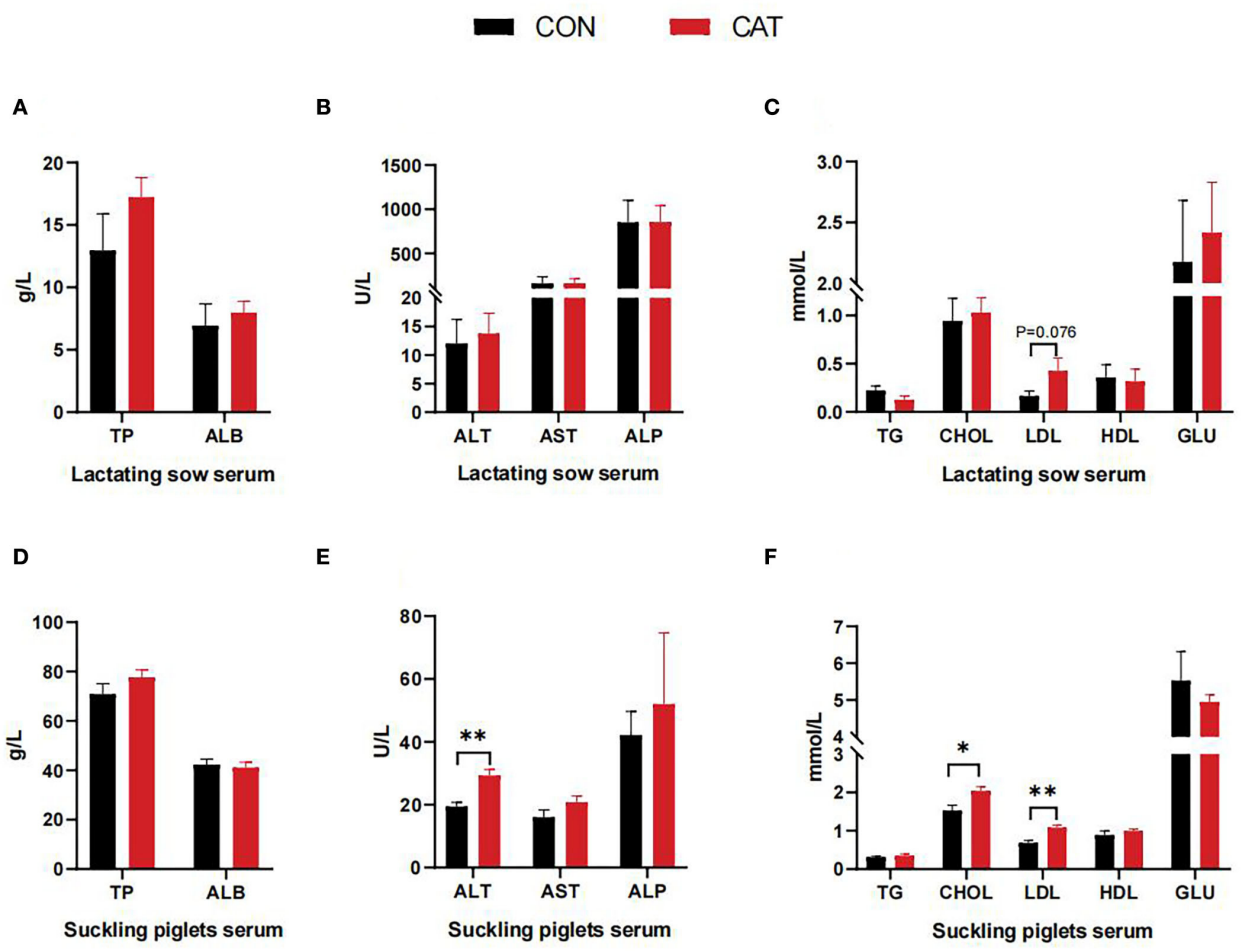
**(A–F)** Serum biochemical indexes. Values are presented as mean ± SEM; *n* = 6, * and **indicate a statistically significant difference by *t*-test at *P* < 0.05 and *P* < 0.01, respectively.

To investigate the effects of maternal CAT supplementation on the lipid metabolism of offspring, we evaluated the related serum biochemical index (Figure 1). Compared to the basal diet, CAT tended to enhance the content of LDL in the serum of sows during lactation (*P* = 0.076). Besides, the level of ALT, CHOL, and LDL in the serum of suckling piglets was increased (*P* < 0.05) in the CAT group as compared with the CON group.

### Serum antioxidant content

**Figure 2:**
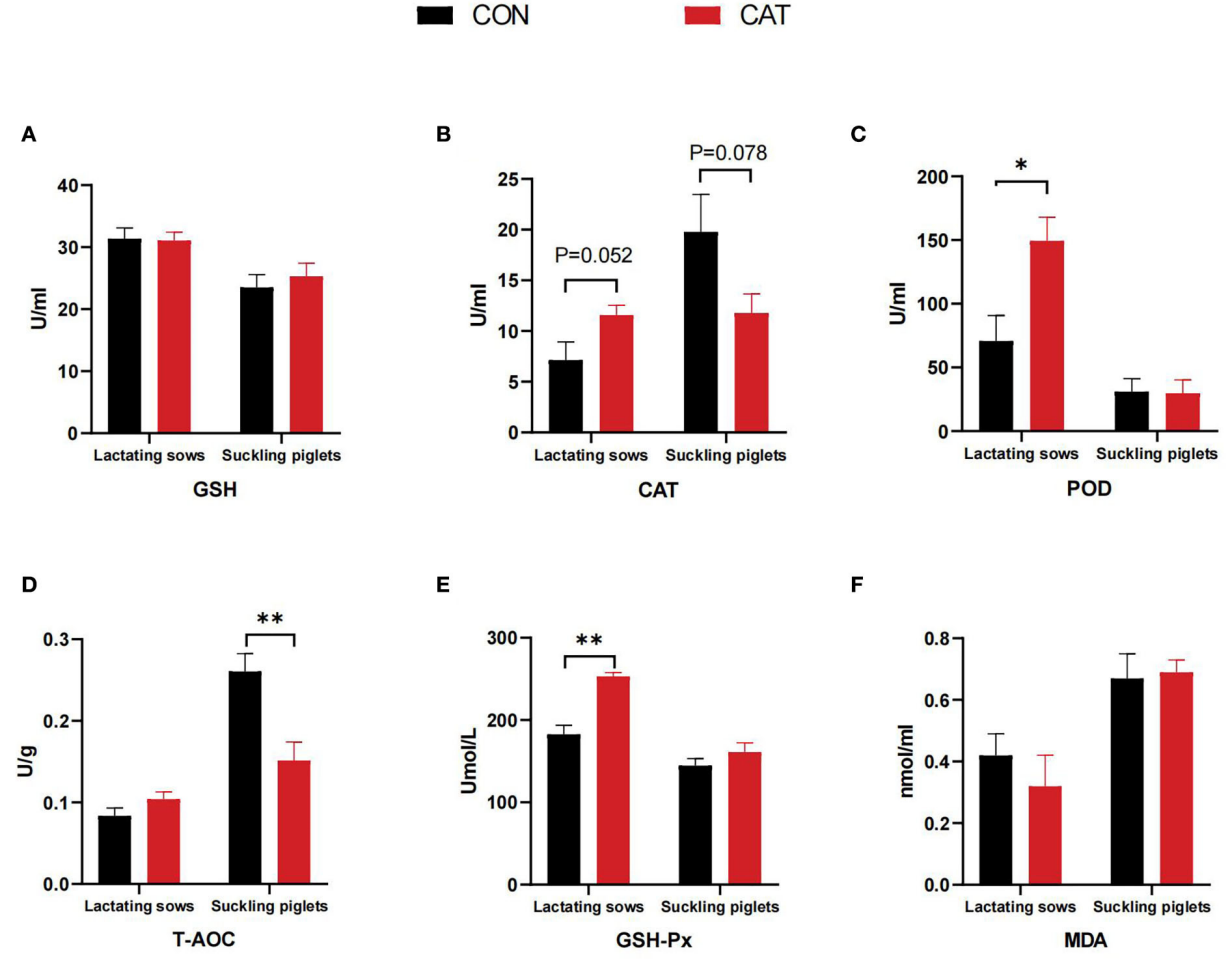
**(A–F)** Serum antioxidant content of lactating sows and suckling piglets. Values are presented as mean ± SEM; * and **indicate a statistically significant difference by *t*-test at *P* < 0.05 and *P* < 0.01, respectively. GSH, glutathione; POD, peroxidase; CAT, catalase; GSH-Px, glutathione peroxidase; T-AOC, total antioxidant capacity; MDA, malonaldehyde.

As shown in Figure 2, maternal CAT supplementation affected the serum antioxidant parameters of lactating sows and their offspring. The serum POD and GSH-Px activities were significantly increased (*P* < 0.05), and the serum CAT activity tended to increase in lactating sows (0.05 < *P* < 0.10) in the CAT group when compared with the CON group. In addition, CAT addition to sows' diet could significantly decrease (*P* < 0.05) the T-AOC level and tended to decrease (0.05 < *P* < 0.10) the CAT activity in the serum of suckling piglets.

### Plasma fatty acid profiles

**Figure 3:**
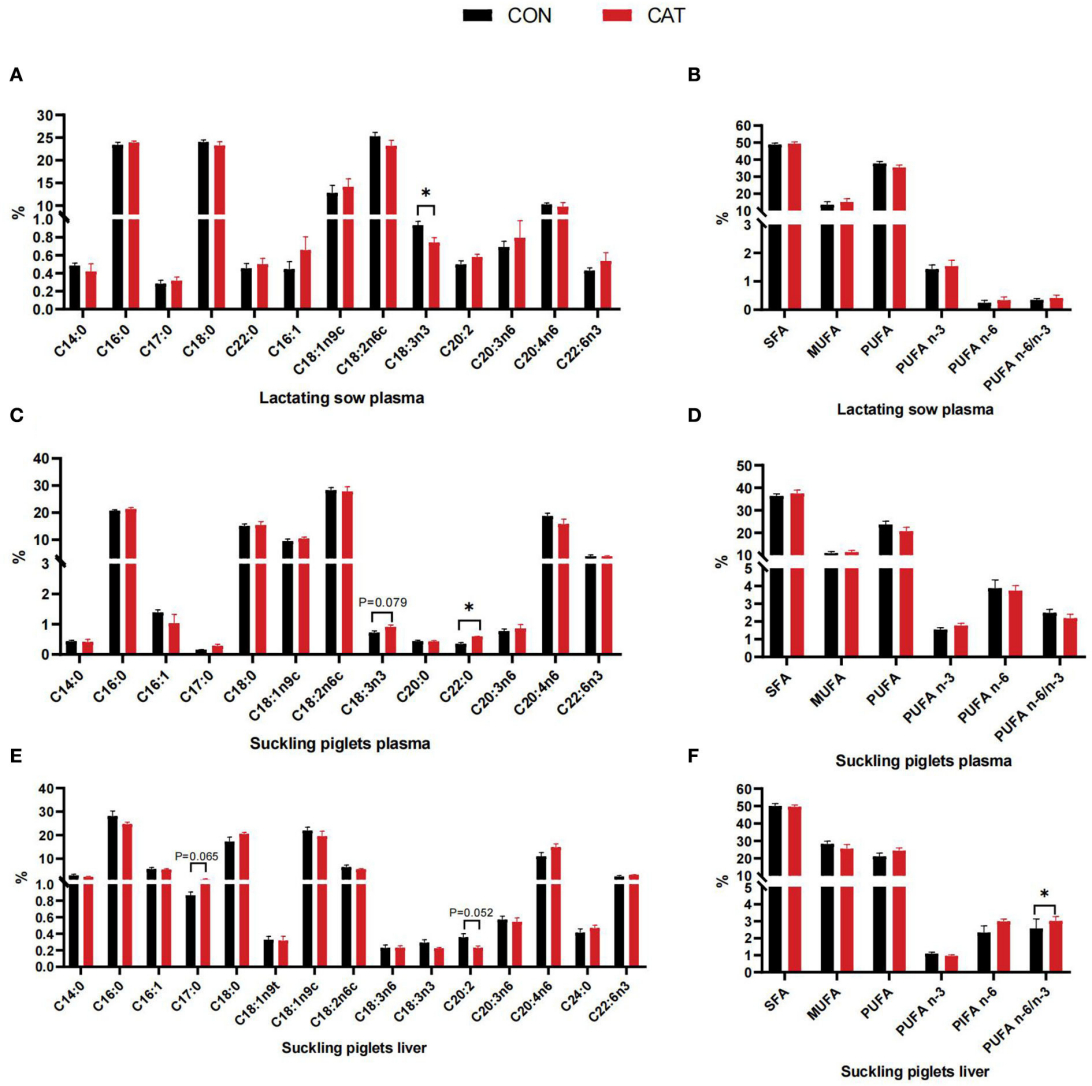
**(A–F)** Plasma and hepatic fatty acid profiles of lactating sows and suckling piglets. Values are presented as mean ± SEM; SFA, saturated fatty acid (including C14:0, C16:0, C17:0, C18:0, C20:0, and C22:0); MUFA, monounsaturated fatty acids (including C16:1, C18:1n9t, C18:1n9c, and C20:1); PUFA, polyunsaturated fatty acids (including C18:2n6c, C18:3n3, C20:3n6, C20:4n6, and C22:6n3); *indicate a statistically significant difference by *t*-test at *P* < 0.05.

The fatty acid profiles of lactating sows and suckling piglets are shown in Figure 3. The results demonstrated that C18:3n3 (α-linolenic acid) content was significantly decreased (*P* < 0.05) in lactating sows' plasma while it tended to increase (*P* = 0.079) in suckling piglets' plasma from the supplemented group. Besides, suckling piglets in the CAT group had a significantly increased plasma concentration of C22:0 (*P* < 0.05) ceramides than in the CON group.

### The mRNA level related to antioxidant enzymes

**Figure 4:**
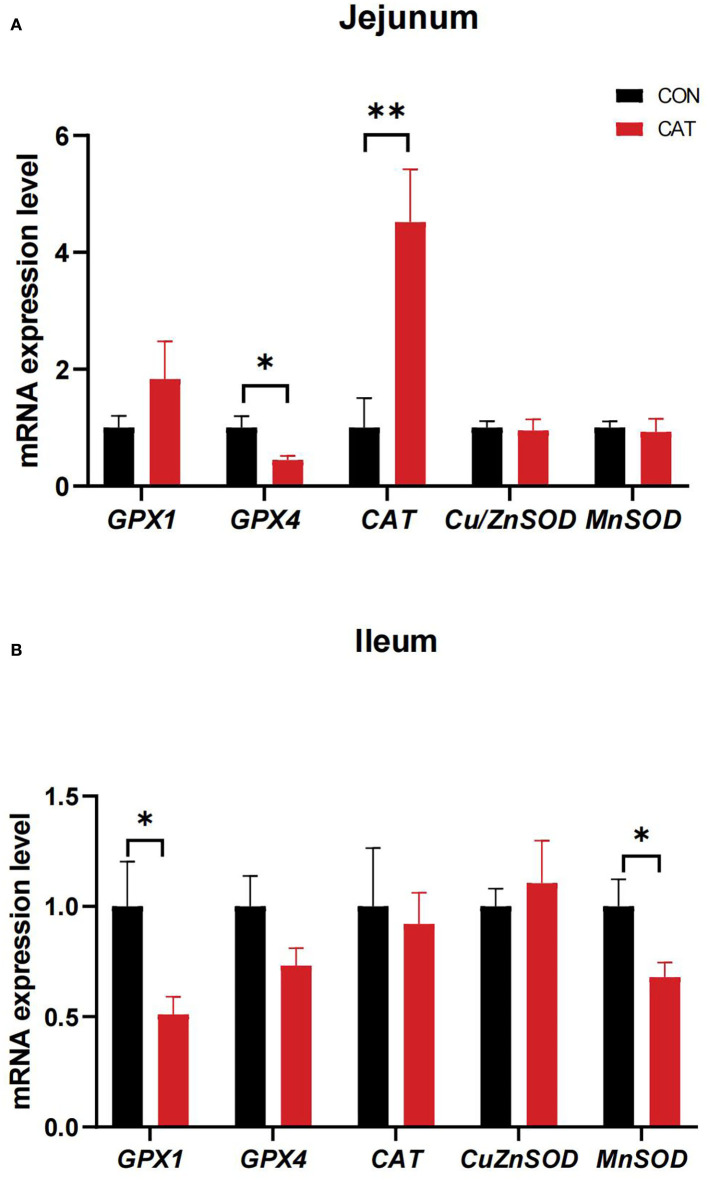
**(A,B)** The mRNA expression of genes related to intestinal antioxidant enzymes of suckling piglets. Values are presented as mean ± SEM; *n* = 6, * and ** indicate a significant difference at *P* < 0.05 and P < 0.01, respectively. GPX1, glutathione peroxidase 1; GPX4, glutathione peroxidase 4; CAT, catalase; Cu/ZnSOD, superoxide dismutase [Cu/Zn]; MnSOD, superoxide dismutase [Mn].

To further evaluate the effects of maternal CAT supplementation on intestinal antioxidant activity, we detected the mRNA expression of intestinal antioxidant enzymes (Figure 4). The results showed that maternal CAT supplementation could significantly downregulate the jejunal mRNA expression of *GPX4* (*P* < 0.05) while upregulating the mRNA expression of *CAT* (*P* < 0.01). In addition, the ileal mRNA levels of *GPX1* and *MnSOD* were significantly decreased (*P* < 0.05).

### The mRNA level related to fatty acid metabolism

**Figure 5:**
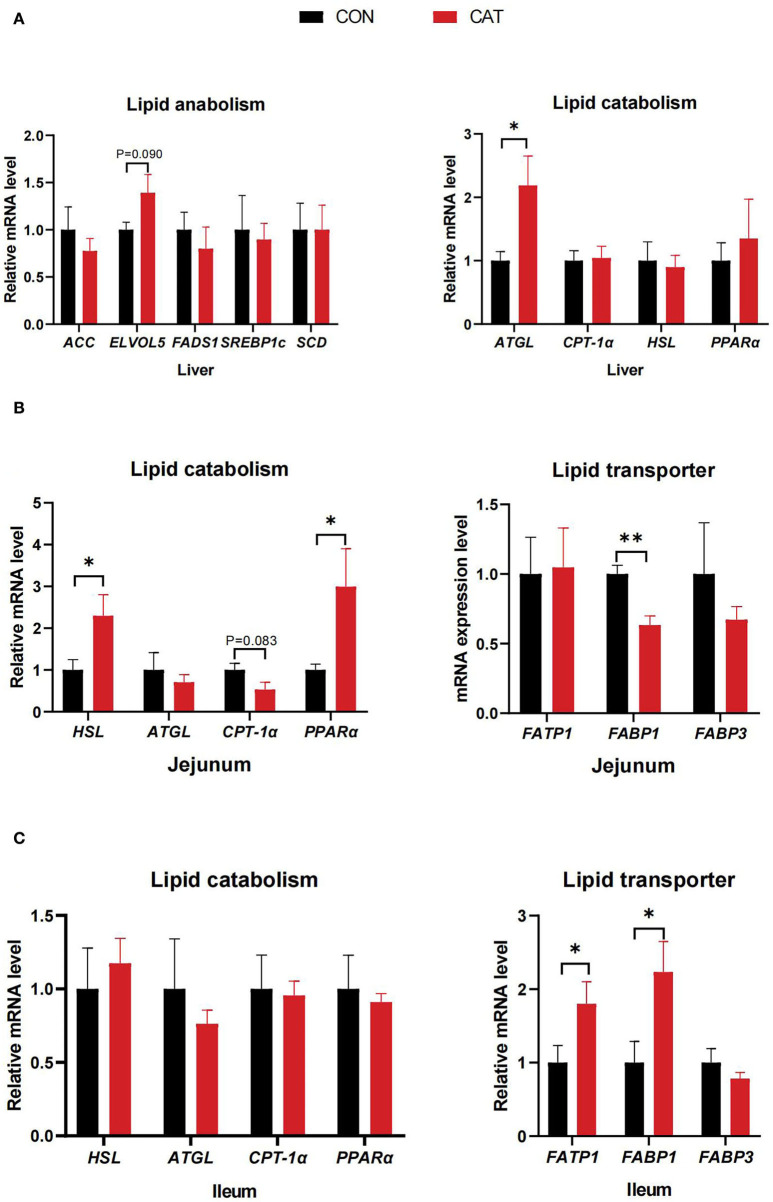
**(A–C)** The mRNA expression of genes related to lipid metabolism of suckling piglets. Values are presented as mean ± SEM; *n* = 6, * and **indicate a significant difference at *P* < 0.05 and *P* < 0.01, respectively. ACC, acetyl-CoA carboxylase; ELOVL5, fatty acid elongase 5; FADS1, fatty acid desaturase 1; SREBP1c, sterol regulatory element-binding protein 1c; SCD, stearoyl coenzyme A desaturase; ATGL, adipose triglyceride lipase; CPT-1α, carnitine palmitoyltransferase 1α; HSL, hormone-sensitive lipase; PPARα, peroxisome proliferator-activated receptor α; FATP1, fatty acid transport protein 1; FABP1, fatty acid-binding protein 1; FABP3, fatty acid-binding protein 3.

To evaluate the effects of CAT supplementation on lipid synthesis, catabolism, and transport of suckling piglets, we measured the related gene expression in the liver, jejunum, and ileum (Figure 5). The results showed that the mRNA expression of adipose triglyceride lipase (*ATGL*) was significantly upregulated (*P* < 0.05), and the mRNA expression of fatty acid elongase 5 (*ELVOL5*) had an increasing tendency (0.05 < *P* < 0.10) in the liver of the CAT group compared with the CON group. Besides, maternal CAT supplementation significantly upregulated the mRNA expression of *HSL* and *PPAR*α (*P* < 0.05), while the mRNA level of fatty acid binding protein 1 (*FABP1*) was significantly decreased and carnitine palmitoyl transferase 1 (*CPT-1*α) expression tended to decrease (0.05 < *P* < 0.10) in the jejunum. Additionally, the mRNA level of fatty acid transport protein 1 (*FATP1*) and *FABP1* was significantly upregulated (*P* < 0.05) in the ileum.

## Discussion

Catalase is a multifunctional enzyme in animals, with widespread adoption in medical, food, animal, and other industries (21, 22). Currently, increasing attention has been paid to the research of fetal programming; however, few studies have reported the effects of CAT on fetal programming. In this study, we aimed to explore the effects of maternal CAT supplementation during late gestation and lactation on the growth performance, amino acid, and fatty acid metabolism of suckling piglets.

In recent years, CAT activity is proven to be related to the growth and aging in the organism, revealing the strong antioxidant, and detoxification ability and the ability to eliminate or inhibit free radicals, thus protecting cells, antiaging, and prolonging life (23). It was reported that weaned pigs fed with 2,000 mg/kg exogenous CAT had higher growth performance and antioxidant capacity (14). The results of this study first revealed that maternal CAT supplementation increased the BW at day 14 of the nursing period and ADG of suckling piglets, despite the similarity of BW at day 7 of the nursing period of piglets between the CON and CAT groups. This may depend on the time length of supplementation with CAT, as CAT supplemented from late gestation may require a certain amount of nutrient reaction time to affect the performance of sows and piglets. Boosted catabolism in sows during pregnancy and lactation can put stress on their health status and metabolism more easily, which may further affect milk production and litter weight gain (24). Under normal circumstances, sows have their own antioxidant defense mechanism, which is mainly composed of antioxidant enzymes (SOD, CAT, GSH, and GSH-Px) and non–enzymatic antioxidants (vitamins, enzyme constituents, etc.,) (23). But, the sows during gestation and lactation are considerably easier to experience oxidative stress, which amounted to scarce antioxidative ability. It is well–established that supplementation with natural antioxidative activities during late pregnancy and lactation could benefit the performance of sows and piglets (22). In this study, no differences in the nutritional composition of colostrum were found, but the milk yield was significantly increased between the CON and CAT groups. Then, we believed that CAT supplementation may alleviate their oxidative stress and improve health status, thus lessening catabolism to provide themselves with more nutrients to produce milk.

As is well-known, catalase functions by metabolizing hydrogen peroxide into water and oxygen to prevent cellular oxidative damage, including oxidative damage to intracellular macromolecules such as lipid peroxidation. Hydrogen peroxide also participates in intracellular signaling pathways acting as second messengers, indicating that catalase may also be involved in lipid metabolism. According to the previous study, CAT deficiency was associated with an increase in adipose tissue and increased levels of serum TG, suggesting a crucial role for catalase in lipid mobilization (25). To investigate the effects of maternal CAT supplementation on the lipid metabolism of offspring, we first evaluated the related serum physiological and biochemical indexes. This study showed that the sows' serum content of LDL had tended to increase and the piglets' serum content of ALT, CHOL, and LDL significantly increased in the CAT group, which demonstrated that maternal CAT supplementation had an effect in regulating blood lipid. Blood lipids can reflect the lipid metabolism in the body, especially the serum CHOL is released into the blood by the liver, carried by LDL, and transported throughout the body to provide energy for metabolism and vital activities. Therefore, an increase in serum content of CHOL and LDL was required for the development of suckling piglets.

Normally, the antioxidant defense system, including antioxidant enzymes such as SOD, CAT, and GSH, is relied on to defense oxidative stress and damage (26). In this study, the serum POD and GSH-Px activities were significantly increased, and the serum CAT activity tended to increase in lactating sows in the CAT group when compared with the CON group. In addition, maternal CAT supplementation significantly decreased the T-AOC level and tended to decrease the CAT activity in the serum of suckling piglets. This suggested CAT administration to sows' diet could improve the antioxidant ability of lactating sows, thereby further diminishing the oxidative stress of suckling piglets. The antioxidant defense mechanism for piglets during the suckling period is susceptible to stress, resulting in restrained growth (27). Some studies have shown that maternal diet can modulate intestinal health in offspring, at least in part by altering intestinal oxidative status and barrier function (28). To further investigate the ability of CAT supplementation on the antioxidant mechanism of suckling piglets, we measured the gene levels of suckling piglets. We found that maternal CAT supplementation could significantly downregulate the jejunal mRNA expression of *GPX4* while upregulating the jejunal mRNA expression of *CAT*, which may demonstrate maternal CAT supplementation could affect jejunal antioxidant metabolism and focus on regulating CAT expression. In addition, the ileal mRNA levels of *GPX1* and *MnSOD* were significantly decreased, most probably due to the conducive effect of maternal CAT supplementation on oxidative stress reduction in the ileum of suckling piglets.

Medium-chain fatty acids (MCFAs) and long-chain fatty acids (LCFAs) are efficiently absorbed and metabolized for piglet nutrition, which contributes to instant energy and even improves performance in the newborn and suckling piglets (29). We further evaluated the fatty acid profiles in the plasma of sows and piglets and the liver of piglets to find the effects of CAT on fatty acid composition. The C18:3n3 (α-linolenic acid) content was decreased in lactating sows' plasma while increased in suckling piglets' plasma, which may demonstrate the C18:3n3 derived from sows was utilized by piglets, and C18:3n3 serves as a vital *n*-3 polyunsaturated fatty acid (PUFA), could play a role in muscle development (30), and promotes the absorption of vitamins, proteins, minerals, and other nutrients (31). Besides, suckling piglets in the CAT group had higher plasma concentrations of C22:0 ceramides than in the CON group, which was a favorable phenomenon because of the pleiotropic effects of ceramides such as regulating apoptosis, differentiation, proliferation, and stress responses (32).

Catalase has been shown to make impression on the liver, with studies showing the total fatty acids in mouse liver homogenates increase with the catalase enhancement (33), and catalase deficiency could regulate the liver fatty acid accumulation by reactive ROS generation (34). The liver is the main organ for lipid metabolism. Thus, in this study, the hepatic MCFA and LCFA proportions of suckling piglets were analyzed. It is worth mentioning that the C17:0 content tended to increase while the C20:2 content tended to decrease, as our results showed, and several studies confirmed that an increasing content of C17:0 was associated with ferritin, inflammation, and the level of glucose, triglycerides, and insulin (35), and the decreased proportion of C20:0 was related to the synthesis of ceramides (32).

Hepatic lipid metabolism requires a series of key enzymes to be involved in lipid anabolism, lipid catabolism, and fatty acid transporters, including ACC, ELOVL5, ATGL, and FATP1. The changes in MCFA and LCFA content in the liver may reveal the impacts of CAT on hepatic enzymes related to lipid metabolism. It was supported in the past that catalase-deficient mice were susceptible to oxidative stress and in turn, caused the activity change in several enzymes involved in lipid metabolism in the liver (36–38). Our results showed that the suckling piglets' hepatic mRNA expression of *ATGL* was significantly upregulated (*P* < 0.05), and *ELVOL5* expression tended to increase (*P* < 0.10) in the CAT group compared with the CON group, which might have a crucial role in lipid catabolism.

The small intestine is crucial for maintaining systemic energy homeostasis, where lipid metabolism also occurs, among which the jejunum is the predominant place for lipid absorption and the ileum serves as a compensatory uptake place (39). The mRNA level *FABP1* was significantly decreased and *CPT-1*α expression had a trend to decrease in the jejunum, these two indispensable enzymes are involved in lipid catabolism and fatty acid transporter, respectively, suggesting that maternal supplemented with CAT may indicate that the low β-oxidation activity of LCFA is reduced along with the diminished fatty acid transport (20). Besides, maternal CAT supplementation could promote the ileum to absorb residual fatty acids that are not absorbed by the duodenum or jejunum by upregulating the ileal mRNA expression of *FATP1*. In addition, CAT may play a role in cholesterol absorption by upregulating the mRNA expression of *HSL*. And the increased level of *PPAR*α might underline that dietary CAT to sows regulates jejunal fat metabolism of offspring through upregulating the expression of *PPAR*α. Besides, maternal CAT supplementation could promote the ileum to absorb residual fatty acids which are not absorbed by the duodenum or jejunum by upregulating the ileal mRNA expression of *FATP1* and *FABP1*.

## Conclusion

In conclusion, maternal CAT supplementation could increase the milk yield, regulate the fatty acid metabolism, and decrease the oxidative stress of sows; then increase the antioxidant ability and benefit the hepatic lipid metabolism of offspring, thus improving their growth performance during the lactating period. These findings might provide a reference value for the utilization of CAT as a nutritional supplement for mothers from late pregnancy to the lactation period to promote the growth of offspring.

## Data availability statement

The original contributions presented in the study are included in the article/supplementary files, further inquiries can be directed to the corresponding author.

## Ethics statement

The animal study was reviewed and approved by Review Committee of the Institute of Subtropical Agriculture at the Chinese Academy of Science and the Protocol Management.

## Author contributions

The authors confirm their contribution to the study as follows: TZ: formal analysis, writing the original draft, review, and editing of the revised manuscript. BC, LG, FR, GG, and TW: formal analysis, review, and editing of the manuscript. XW: conception of the idea, investigation, review, editing of the manuscript, administration-lead, and supervision-lead. All authors contributed to the article and approved the submitted version.

## Funding

This study was jointly supported by grants from the Tianjin Synthetic Biotechnology Innovation Capacity Improvement Project (TSBICIP-CXRC-031) and the China Agriculture Research System of MOF and MARA (CARS-35).

## Conflict of interest

The authors declare that the research was conducted in the absence of any commercial or financial relationships that could be construed as a potential conflict of interest.

## Publisher's note

All claims expressed in this article are solely those of the authors and do not necessarily represent those of their affiliated organizations, or those of the publisher, the editors and the reviewers. Any product that may be evaluated in this article, or claim that may be made by its manufacturer, is not guaranteed or endorsed by the publisher.
